# Predictors of Occult Chondral Injury Sustained After a Primary Patellar Dislocation

**DOI:** 10.7759/cureus.22516

**Published:** 2022-02-23

**Authors:** Jensen G Kolaczko, Lucas Haase, Matthew Kaufman, Jacob Calcei, Michael R Karns

**Affiliations:** 1 Orthopedic Surgery, University Hospitals Cleveland Medical Center, Cleveland, USA; 2 Orthopedic Surgery, Case Western Reserve University School of Medicine, Cleveland, USA

**Keywords:** orthopedic surgery, sports medicine, osteochondral defect, knee arthroscopy, patella dislocation

## Abstract

Background and objective

Primary patellar dislocations can concomitantly involve osteochondral injuries for which prompt recognition is paramount for joint preservation. These injuries can be missed on radiographs, necessitating MRI examinations. In this study, we aimed to identify patient parameters that correlate with occult osteochondral injuries.

Methods

Patients were retrospectively identified between 2015 and 2020 through a chart review. The inclusion criteria were as follows: patients diagnosed with a primary patellar dislocation with three radiographic views and an MRI of the injured knee. Demographic and radiographic data were evaluated.

Results

A total of 61 patients met the inclusion criteria. There were no statistically significant demographic differences between patients with osteochondral injuries and those without (p>0.05). Seven knees (88%) with an osteochondral lesion and 20 (38%) without had an effusion (p=0.02). There was no association in terms of ligamentous laxity (p=0.49), Caton-Deschamps index (CDI) (p=0.68), sulcus angle (SA) (p=0.68), congruence angle (CA) (p=0.56), and lateral patellofemoral angle (LPFA) (p=0.25) between patients with and without an occult osteochondral injury.

Conclusion

Among the parameter examined, the presence of an effusion was the only one that correlated with the presence of occult osteochondral injury in our cohort.

## Introduction

Patellar dislocations account for 3% of knee injuries and are the second most common cause of traumatic knee hemarthrosis [1,2]. The classic injury mechanism involves knee flexion and a valgus moment, which accounts for as many as 93% of dislocation events [3]. Patellar dislocations can concomitantly involve osteochondral injuries, with reported incidence rates from 5 to 68% [4]. These injuries are known to be more prevalent than usually identified on initial radiographs [5-7]. The chance of sustaining osteochondral injuries with a patellar dislocation is related to factors of knee anatomy, with patients with patellofemoral dysplasia requiring lower energy mechanisms to dislocate and subsequently lower probability of intraarticular injuries. Conversely, in patients without evidence of trochlear dysplasia and with normal alignment, higher energy is required for a patellar dislocation to occur and these patients have higher rates of osteochondral injuries [4,8].

The presence of osteochondral fractures has been consistently shown to affect the progression of osteoarthritis in the patellofemoral joint [9,10]. This progression is likely caused by increased acute inflammation due to the exposed subchondral bone as well as an alteration of stress distribution within the joint [11,12]. For these reasons, the early recognition of osteochondral fractures is paramount for long-term joint preservation. Unfortunately, these injuries can be missed in as high as 68% of initial radiographs [7]. MRI is the imaging modality of choice for assessing the patellofemoral cartilage as well as the medial stabilizing structures including the medial retinaculum and medial patellofemoral ligament [13,14]. Classically, an MRI is obtained after a traumatic patellar dislocation if a large effusion is present on examination [15]. However, to date, no study has attempted to identify which radiographic parameters would prompt an MRI evaluation. In light of this, we conducted this study to identify radiographic and clinical parameters that correlate with occult osteochondral injuries and warrant an MRI after a primary patellar dislocation.

## Materials and methods

After obtaining institutional review board approval from the University Hospitals Cleveland Medical Center (approval no: STUDY20200647), the electronic medical records of patients suffering a patellar dislocation event between 2015 and 2020 were identified by examining the billing database using the International Classification of Diseases, Tenth Revision, Clinical Modification (ICD-10-CM) code S83.0. Inclusion criteria were as follows: patients of all ages diagnosed with a primary patellar dislocation with three radiographic views of the knee (AP/lateral/sunrise or Merchant view) without evidence of loose body or osteochondral injury, and an MRI of the injured knee. Patients were excluded if they were not diagnosed with a patellar dislocation, had multiple dislocations, prior knee surgery, had evidence of loose bodies or osteochondral injury on X-ray, and if X-rays and MRI were unavailable or missing. Demographic data for each patient was recorded via chart review, and this included age, sex, laterality of injury, BMI, number of dislocation events, mechanism of injury, presence of ligamentous laxity as defined by the Beighton score, sporting activity at the time of injury, concomitant knee pathology, and the presence of an effusion. All radiographic images were available electronically and evaluated using Sectra Workstation IDS7 (Sectra AB, Linköping, Sweden). The following data points and measurements were recorded: osteochondral defect or loose body, trochlear dysplasia based on the Dejour classification, Caton-Deschamps index (CDI), sulcus angle (SA), lateral patellofemoral angle (LPFA), and congruence angle (CA). These measurements were made in accordance with previously described and validated techniques [16-18]. Two independent reviewers evaluated subjects' X-rays, clinical notes, and MRIs for evidence of loose bodies and osteochondral injuries and measured each angle, and intraclass correlation coefficients (ICCs) were calculated. An ICC <0.4 was considered poor, 0.4-0.75 was deemed fair to good, and >0.75 was considered excellent [19,20].

Descriptive and comparative statistics were utilized for data analysis. For continuous variables such as CDI, SA, LPFA, CA, BMI, and age, comparisons were made using the Student’s t-test. Qualitative variables such as laterality, presence of an effusion, presence of ligamentous laxity, trochlear dysplasia, and patella alta were compared using a chi-squared test. A p-value <0.05 was considered statistically significant. All data were analyzed using SPSS Statistics version 24 (IBM, Armonk, NY). All data were stored electronically in a secure fashion in accordance with the IRB guidelines.

## Results

After examining the billing database, 331 knees in 329 patients were identified; 270 of these patients were excluded from participation in the study based on the criteria outlined above. After the exclusion, there were 61 patients available for review with first-time patellar dislocations. The average age of patients at the time of injury was 16.8 years; 24 (39%) dislocations events involved the right knee, while 37 (61%) involved the left knee. The average BMI was 26.6 kg/m^2^. Eight knees (13%) had osteochondral injuries, while 53 (87%) did not. There were no statistically significant demographic differences between patients with osteochondral injuries and those without (p>0.05). Seven knees (88%) with osteochondral injuries had an effusion on exam and evaluation of radiographs while 20 (38%) of the knees without an osteochondral defect had an effusion (p=0.02). There was no association between ligamentous laxity and the presence of an osteochondral defect (p=0.49). Table 1 presents the patient characteristics.

**Table 1:** Patient characteristics P≤0.05 is considered statistically significant; value in bold indicates significant value SD: standard deviation

Variables	Chondral defect (n=8)	No chondral defect (n=53)	P-value
Age (years), mean± SD	16.13 ± 2.29	16.87 ± 8.65	0.81
Body mass index (kg/m^2^), mean± SD	27.69 ± 9.74	26.41 ± 7.98	0.68
Method of injury, n	Non-contact: 7	Non-contact: 47	0.92
Laterality, n	Right: 3	Right: 21	0.91
	Left: 5	Left: 32	0.91
Effusion present, n (%)	7 (87.5%)	20 (37.7%)	0.02
Ligamentous laxity, n (%)	2 (25%)	5 (9.4%)	0.49

Radiographic measurements demonstrated an average CDI of 1.19 in patients with osteochondral injuries and 1.16 in patients without (p=0.68). Patients with osteochondral injuries had an SA of 126.3° and CA of 10.8° compared to 128.1° and 12.0° in patients without osteochondral injury (p=0.68 and 0.56 respectively). There was no association between LPFA and the presence of osteochondral injuries (p=0.25). None of the radiographic measurements demonstrated a significant association with osteochondral injury (Table 2). The measurements performed in this study did demonstrate excellent inter-rater reliability with the SA ICC (0.96), the CA (0.89), the LPFA (0.91), and the CDI (0.97).

**Table 2:** Radiographic characteristics P≤0.05 is considered statistically significant SD: standard deviation

Variables	Chondral defect (n=8)	No chondral defect (n=53)	P-value
Caton-Deschamps index, mean± SD	1.19 ± 0.11	1.16 ± 0.17	0.68
Sulcus angle, mean± SD	126.34 ± 12.9	128.13 ± 11.1	0.68
Congruence angle, mean± SD	10.8 ± 4.6	12.0 ± 5.1	0.56
Lateral patellofemoral angle, mean± SD	26.9 ± 11.9	22.6 ± 9.4	0.25
Presence of trochlear dysplasia, n (%)	0 (0%)	6 (11.3%)	0.72
Patella alta, n (%)	1 (12.5%)	11 (20.8%)	0.94

## Discussion

This study examined 61 patients with primary patellar dislocation events with the aim of revealing a relationship between osteochondral injuries and the demographic, clinical, and radiographic features of these patients. Upon analysis, the presence of an effusion was the only factor that showed a statistically significant association with an osteochondral injury. The incidence rate of osteochondral injuries after a primary patellar dislocation was 13%. This is about half the rate noted in a previous systematic review [4]. To our knowledge, this is the first study to analyze radiographic and demographic parameters to predict osteochondral injuries in first-time patellar dislocation events.

Certain patient factors have been implicated in the risk of sustaining a patellar dislocation. Female sex and obesity have been linked to a propensity for sustaining a patellar dislocation [3]. However, the current study could not validate this, since there was a similar or identical number of female and male patients in both the outcome groups. In addition, the average BMI was in the “overweight” category, not “obese”. Laterality and non-contact vs. contact injury also did not show any relation to the presence of an osteochondral injury. The male-to-female ratio of our sample population was 39:61%. The female predominance seen here has been previously described in the literature [3,11,21]. Likewise, it has been shown that ligamentous laxity can predispose individuals to patellar instability [21]. However, no statistically significant relationship between ligamentous laxity/hypermobility and osteochondral injuries was identified in the current study.

As mentioned, individuals with certain radiographic parameters have an increased risk for patellar instability. Trochlear dysplasia and patella alta lead to abnormal knee kinematics that results in an increased risk of patellar instability due to a lack of containment [4,8]. Previous studies have examined the incidence of articular cartilage injuries after patellar dislocation and noted that patients with normal patellar stability had a 2.5 times greater risk of sustaining an osteochondral injury when compared to those with abnormal features [22]. In contrast, the current study did not find any statistically significant relationship between abnormal radiographic parameters of the knee and the absence or presence of an osteochondral injury as identified on MRI evaluation. This lack of correlation reinforces the findings of two recent MRI-based studies that examined anatomic patellar instability parameters and their relation to intraarticular injury patterns after a primary lateral patellar dislocation. These studies found that underlying risk factors for classic lateral patellar instability did not predict injury patterns and classic dysplastic features did not decrease the likelihood of injury to the articular surface [23,24]. Previous studies have shown that the presence of a large hemarthrosis could be indicative of an osteochondral injury [15]. Similarly, if an effusion is present, it may represent a hemarthrosis, which has been shown to increase the risk for an underlying osteochondral injury. The presence of an effusion should prompt the surgeon to obtain an MRI to evaluate for an occult osteochondral injury not found on radiographs [8]. The authors are in agreement with this sentiment since 87.5% of those with osteochondral injuries had an effusion on X-ray or clinical exam.

There are several limitations to the current study. Firstly, this was a retrospective review with patients identified by ICD-10 codes, and hence we may have failed to include some of the patients by using incorrect codes. Second, the sample size was small and therefore some of the in-question variables did not reach statistical significance due to inadequate power. Thirdly, due to its retrospective nature, the study relied on the operative reports and the surgeons’ descriptions of the osteochondral injuries, which are inherently prone to interpretation bias. The presence of an effusion on a clinical exam may also be associated with observer bias, though previous studies have demonstrated good inter and intraobserver reliability for the assessment of a knee effusion [25]. Lastly, this study only included patients who sustained a first-time patellar dislocation. Those individuals who presented due to recurrent patellar instability either with or without surgery were excluded. We concede that this decreased the sample size but also limited a potential confounding variable. In addition, we note the possibility of selection bias since all included patients had an MRI after a primary patellar dislocation. This may have led to the selection of patients in whom the evaluating practitioner had concerns for significant injury. There may have been more first-time dislocations that did not receive an MRI, and hence the condition may have a higher incidence rate than what we observed.

## Conclusions

This is the first study to analyze demographic, clinical, and radiographic parameters to predict the presence of an occult osteochondral lesion after a primary patellar dislocation. A patellar dislocation with concomitant osteochondral injury or loose body can be devastating and requires early surgical intervention. Prompt identification is paramount to reduce and prevent future damage to the knee joint. We attempted to identify radiographic and demographic parameters that correlate with occult injuries and warrant an MRI after a primary patellar dislocation. Upon statistical evaluation, none of the demographic, clinical, or radiographic variables examined was found to have a significant association with the presence or absence of a knee osteochondral lesion, except for the presence of an effusion. Therefore, we recommend that surgeons obtain an MRI for all patients with knee effusions after a patellar dislocation, even if initial X-rays are negative for pathology. More high-quality research is needed to further elucidate the risk factors that may help clinicians to predict osteochondral injuries after patellar dislocations.
